# Phototactic Responses to Ultraviolet and White Light in Various Species of Collembola, Including the Eyeless Species, *Folsomia candida*

**DOI:** 10.1673/031.007.2201

**Published:** 2007-04-12

**Authors:** Gregory L. Fox, Catherine A. Coyle-Thompson, Peter F. Bellinger, Randy W. Cohen

**Affiliations:** Department of Biology, California State University, Northridge, 18111 Northoff St., Northridge, CA 91330-8303

**Keywords:** Isotomidae, photoreceptors, SEM, ommatidium, behavior

## Abstract

Previous observations have indicated homology in the cellular components between collembolan eyes and the compound eyes of insects. However, behavioral or physiological studies indicating similarities in function are lacking. Collembolan eyes were examined from three species in the Family Isotomidae using scanning electron microscopy. Collembolan eyes are arranged dorsally and laterally on each side of the head in two species, *Proisotoma minuta* with eight eyes on each side of the head and *Folsomia similis* with one eye on each side of the head. In both of these species the eyes were located just posterior to the postantennal organ. In *Folsomia candida*, no external eye structures were detected. These three species were assayed for a series of behavioral preferences using ultraviolet (UV), white light and dark, and temperature conditions. The tests demonstrated that over 76% of all three species, including the eyeless *F. Candida*, chose white over UV light, over 69% preferred dark over UV, and over 77% favored dark over white light. The results demonstrated that all three species detect both UV and white light and avoid it, preferring cool, dark habitats. From the results of this study, it is hypothesized that *F. candida* may, in fact, be only “lensless” and may be able to detect light by having internal, non-ocular photoreceptors. Further histological studies are needed to investigate this possibility.

## Introduction

Among Collembola, the number of eyes varies, ranging from eight eyes on each side of the head (8+8 arrangement), to the absence of eyes (0+0). This variation in eye number is common within families of Collembola including the Isotomidae (Bellinger and Christiansen 1980). Regardless of numbers of eyes, the morphology of the eyes of Collembola appears to contain all the same cellular components as the Crustacea and the Insecta. In the studies by Paulus (1970a, 1972a) and Barra (1971), collembolan eyes have been described as being ommatidium-like, containing corneagen cells, primary pigment cells, crystalline cone, four Semper cells and eight rhabdomeres. There appear to be eight retinula cells as is also the case with insects and Crustacea (Gupta 1979; Chapman 1998).

In contrast, Christian and Schaller (1982) originally hypothesized that the collembolan eye is in reality primitive in structure and further suggested that they are structurally more like the simple eyes, or stemata, of holometabolous insect larvae. They described the reduction in the number of eyes in the cave-dwelling species, *Bonetogastrura cavicola*. Their study concluded from external ultrastructural studies that the eyes of this particular species were more like insect ocelli due to the reduction in retinula cells, cornea, crystalline cones, and fusion of the eye structures. In the genus *Orchesella*, the primary pigment cells lack both pigment granules and cytoplasmic structures such as mitochondria or endoplasmic reticulum, and therefore were considered degenerate (Paulus 1970b).

If collembolan eyes are homologous to the stemmata of insects do they detect and discriminate between both ultraviolet (UV) and white light as insect stemmata do? Many diurnal insects use white light to direct themselves to food sources, while others, such as ants and cockroaches that normally dwell in the dark, detect UV light and use this sensory information to orient themselves away from the source (Wigglesworth 1972). Since Collembola are ubiquitous decomposers, it would be an important asset for Collembola as soil-dwelling hexapods to detect both UV and white light so that they might be able to orient themselves, gaining directional reference (Hägvar 1995).

In a study by Schaller (1970), the responses to different light conditions were observed, and it was notated that Collembola moved toward dark vertical objects in a white arena. The Collembola appeared to detect the movement of conspecifics, either by moving to a congregation of them, or avoiding another Collembola in a one-on-one situation. Salmon and Ponge (1998) showed that the collembolan, *Heteromurus nitidus*, orients itself using white light. Hägvar (1995) demonstrated that the collembolan, *Hypogastura socialis*, appears to detect the angle of the sun before using the furcula to jump. The author suggested that this species has the ability to detect the polarization angle of light before crossing open spaces, perhaps using light for directional cues.

The purpose of this study was to examine the photoreceptor ability of the collembolan eye by identifying the phototactic responses influenced by certain light and temperature conditions. The eye ultrastructure of three Collembolan species was examined: *Proisotoma minuta*, with eight pairs of eyes, *Folsomia similis*, with one pair of eyes, and a presumed negative control, *Folsomia candida* in which eyes are absent. If collembolan eyes are structurally homologous with the insect compound eye then, functionally, they should be able to distinguish among UV, white light, and darkness. In this part of the study, the three species were then tested for choices among UV light, white light and the absence of light.

## Materials and Methods

### Collembola stocks

Three species of Collembola, *F. candida, F. similis*, and *P. minuta*, from the Family Isotomidae were used for this study. *P. minuta* were collected from loose top-soil in Tujunga, CA., USA. Cultures of *F. Candida* and *F. similis* were obtained from Dr. Ken Christiansen and Mr. Tom Smith, respectively. Collembola were cultured in plastic, screw top containers (60 mm × 30 mm) with a Plaster of Paris/charcoal substrate. Each culture was provided weekly with 0.5–1.5 ml of distilled H_2_O and three granules of active yeast (Red Star®) per ten individuals. Cultures were kept in an incubator at 22±1° C. Cultures were kept on a 14:10 (dim light: dark) photoperiod.

### Scanning electron microscopy (SEM)

A method devised by (Coyle-Thompson and Banerjee 1993) for preparing *Drosophila* for scanning electron microscopy (SEM) was modified to view the collembolan eyes. The Collembola were placed in a freezer at -20° C for 20 min. The cold temperature stopped their movement and froze them with their heads extended in proper orientation. They were then fixed in 70% ethanol for at least seven days. The specimens were later transferred to a 9:1 PEMS/formaldehyde solution for 2 hours followed by dehydration in a series of increasing percentages of ethanol. Critical point drying was accomplished by placing specimens in hexamethydizalisane for 5 min. After desiccation, they were mounted on aluminum SEM mounts, sputter-coated with gold, and stored before use. The external ultrastructure of the collembolan head was observed on a JOEL Scanning Electron Microscope (model #JSM-5400). Ten individuals of each species were processed and imaged.

**Figure 1.**
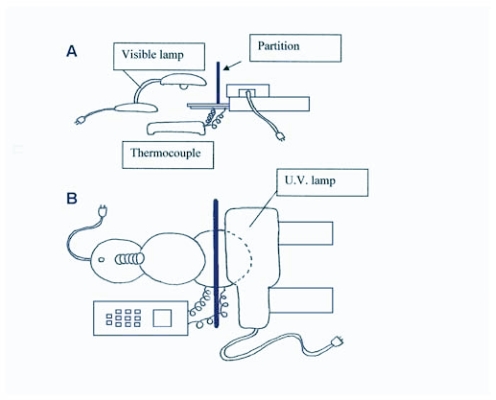
Side (A) and top (B) views of the behavioral assay setup.

### Behavioral assays - Set-up

Choice-testing chambers were made from clear plastic 150 mm diameter, circular Petri dishes. The chamber bottoms were filled (0.5 cm depth) with the same plaster/charcoal mixture used in the culture jars. A dual channel thermocouple (Barnant® model 600–1040) probe was inserted through a hole in the side of each chamber.

A 60 watt lamp (7 lux) was used for a white light source, a multiband, ultraviolet (UV) mineral light (AMP model # UVGL-55; 305 nm wavelength; 3 lux) was used for the UV source, and a 25 watt fluorescent lamp (6 lux) was used as a cool light source. Using a modified technique described by Salmon and Ponge (1998), the dark half of the chamber was produced by covering the Petri dish lid with a 0.64 cm thick, half moon shaped piece of black acrylic that blocked all light. Before beginning any UV light assays, strips of paper impregnated with ethidium bromide (EtBr) were placed under the blackened half of the Petri dish to detect ultraviolet rays. An assay was not conducted if the EtBr strips detected ultraviolet light. Prior to all white light assays, strips of Kodabrome II RC F4 Glossy coated photosensitive paper were placed under the blackened half of the Petri dish to detect white light. Again, no assay was conducted if the photosensitive paper detected white light under the dark half of the Petri dish. All behavioral assays were performed at night in a darkened room at room temperature 22 ± 0.5° C. Figure 1 illustrates the setup used for all behavioral assays.

### Behavioral assays - Experiments

To determine responses to different types of light, Collembola were tested for a choice between: 1.) UV light and white light, 2.) UV light and dark, 3.) white light and dark. Prior to the start of each experiment, separate groups of Collembola were selected at random from each colony and placed in individual testing chambers. For each choice situation, three different populations of each species were removed from their culture dishes and were assayed for their phototropic responses. Each population group was tested 3–10 times before they were removed from the testing chamber. The testing chamber bottoms were always rotated after each trial so that the other dish half would be exposed to the alternate light condition, eliminating any possible preferences for a particular side of the dish. The trials of each of the particular behavioral assays lasted for 30 min. Each population group was rested between trials in the dark room for at least five minutes before retested. At the end of each assay, all experimental collembolans were removed from the dish and discarded before another group was selected.

*F. candida* (n = 34–57 per trial), *F. similis* (n = 24–61 per trial) and *P. minuta* (n = 26–71 per trial) were used for a choice between UV light and white light, replicated 3–10 times. In another three sets of trials, different populations of *F. candida* (n = 33–73 per trial), *F. similis* (n = 44–59 per trial) and *P. minuta* (n = 49–66 per trial) were replicated 3–10 times for a choice between UV light and dark. To test for a choice between white light and dark, 3–10 different trials were performed with three different populations of *F. candida* (n = 33–63 per trial), *F. similis* (n = 28–47 per trial) and *P. minuta* (n = 41–58 per trial). After each trial, the number of Collembola on each side of the Petri dish was counted to determine how many had chosen UV light or white light, UV light or dark, and white light or dark. The average number and percentage of 3–10 trials were recorded as the population value.

### Behavioral assays - Temperature effects

A slight temperature difference was detected between the UV light, white light and dark choice assays. Prior to the start of this set of experiments, the temperature in each chamber was measured: UV chamber temperature 24.8 ± 0.5° C; white light chamber temperature 28.4 ± 0.5° C; dark chamber temperature 21.9 ± 0.5° C. To examine temperature as a possible factor influencing the movement of Collembola during the light assays, ten trials (with three separate populations of each species) were performed to determine if Collembola made a choice between warmer temperatures (30 ± 1° C) or cooler temperatures (22 ± 0.5° C). The number of Collembola used from each species was as follows: *F. candida* (n = 44–66 per trial), *F. similis* (n = 41–52 per trial) and *P. minuta* (n = 37–50 per trial). They were tested again in 30 min intervals in each trial.

For the warm side of the choice-testing chamber, a piece of 0.64 cm thick, half-moon shaped piece of black acrylic wrapped with black tape covered half of the 150 mm diameter, circular Petri dish. A 60 watt lamp was illuminated above the blacked-out side of the choice-testing chamber and used as a heat source. To see if light was blocked and only heat was radiated, strips of photosensitive paper were again placed inside the chamber. For the cool side of the choice-testing chamber, a piece of styrofoam was used to cover half the lid of the Petri dish. The styrofoam layer on the cool side of the choice-testing chamber was then covered with aluminum foil. A 0.64 cm thick × 15 cm high × 36 cm wide piece of black acrylic was used as a partition that rested atop the Petri dish cover to help block heat from reaching the cool side. A dual channel thermocouple with J type leads (Barnant ® model #600–1040) was used to confirm the temperature differences on each side of the Petri dish. The bottom of the Petri dish (choice-testing chamber) was again rotated after each trial to alternate the sides of the dish being exposed to either warmer temperatures or cooler temperatures.

### Cool light versus cool dark

To test the possibility that the Collembola were moving away from the heat emanating from a light source, *F. candida* (n = 20–27 per trial), *F. similis* (n = 19–22 per trial), and *P. minuta* (n = 17–22 per trial) were tested for a choice between a “cool light” and “cool dark” source. The behavioral assays for the “cool light” versus “cool dark” were performed at night in a darkened room. A hand-held 20 watt, 6 lux, fluorescent light was in used as a cool light source. No light source was used for the cool dark side.

A 0.64 cm thick × 15cm high × 36 cm wide piece of black acrylic partition separated the cool light side from the dark side of the Petri dish. The dual channel thermocouple with J-type leads was used to determine that the temperature of each side of the dish was similar to room temperature 22 ± 0.5° C. In this assay, three populations of each species were choice-tested in 3–10 separate trials. Each trial lasted 30 min, and the dish was once again rotated before each trial so that the cool light and cool dark sides of the choice-testing chamber would be different for each trial.

### Statistics

A *χ*^2^ test was used to analyze the results of each of the behavioral assay trials of each species of Collembola used in the trials. The data were tested against an assumed 50:50 distribution. First, heterogeneity *χ*^2^ analysis was performed to see if the population data (3–10 replicates per population group) could be pooled as an individual result among the population trials of each species. If the data were pooled, then a *χ*^2^ analysis was performed on the pooled group data involving the three separate populations. The results were deemed significant at p<0.05.

**Figure 2.**
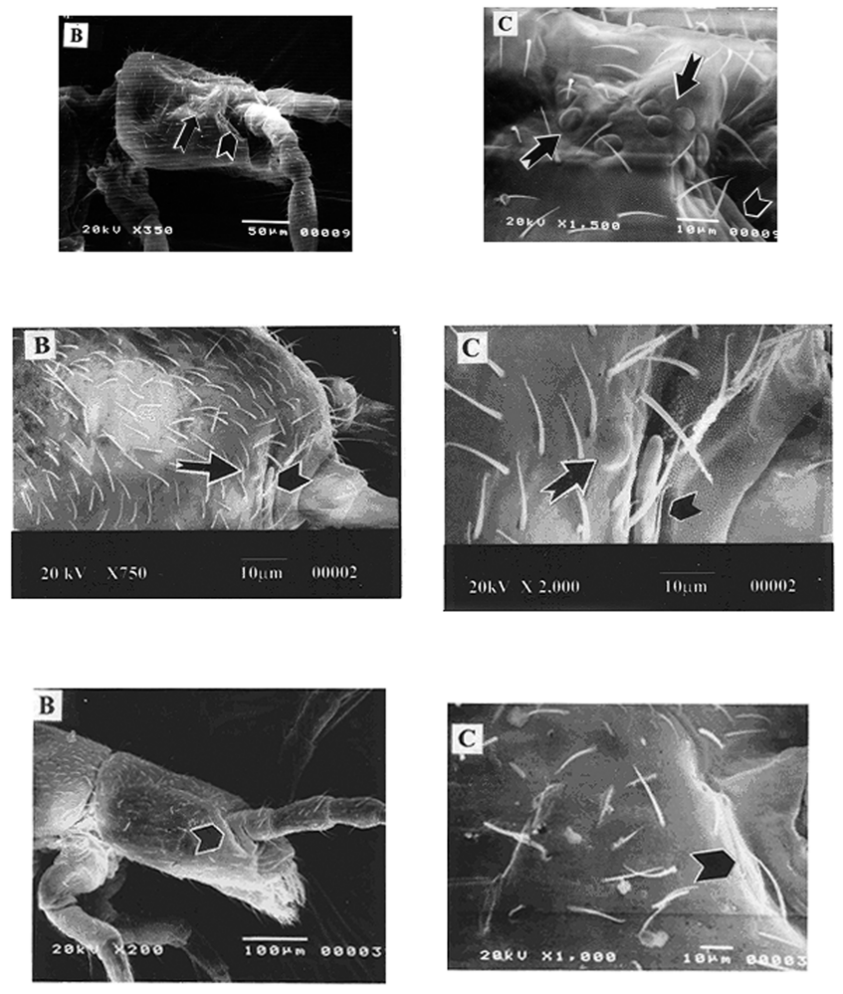
A). Scanning electron microscope photographs of *Proisotoma minuta*. The head is shown is on the left at 350X magnification. The notched arrow indicates the eye patch and the chevron indicates the post-antennal organ (PAO). A high magnification of the head is shown on the right at 1,500X magnification. he notched arrows indicate the eyes and the chevron notates the PAO. B) Scanning electron microscope photographs of *Folsomia similis*. The head is shown on the left at 750X magnification. The notched arrow points to the eye, and the chevron notates the PAO. A high magnification of the head is shown on the right at 2000X magnification. The notched arrow points to the eye and the chevron indicates the PAO. (C) Scanning electron microscope photographs of *Folsomia candida*. The head is shown on the left at 350X magnification with the notched arrow indicating the PAO. High magnification of the head is shown on the right at 1000X magnification, illustrating the lack of external eye structures. For all photomicrographs, anterior is to the right and dorsal is towards the top of the page.

## Results

### Scanning electron microscopy

Scanning electron microscope (SEM) photographs of *P. minuta*, a species with eight pairs of eyes, are shown in Figure 2A. The location and the pattern of arrangement of the eyes are readily seen in this species. The entire head showing the structure of the eyes, and the group of eight eyes was observed and is located just posterior to the post-antennal organ (Figure 2A). In this species, all eight eyes were large, convex and circularly arranged dorsal to lateral.

The structure and the location of the eyes of *F. similis*, the species with one pair of eyes, is shown in Figure 2B. The eyes in all the examined specimens of *F. similis* are located just posterior to the post-antennal organ. Upon closer examination, the eye facet is similar in size and structure to those seen in *P. minuta*.

The facets in both *P. minuta* and *F. similis* appear to have a convex circular lens with an external hexagonal cuticle layer very similar in morphology to the hexagonal pattern of the collembolan exoskeleton. This correlates with morphological findings and descriptions by Bellinger and Christiansen (1980).

SEM photographs of *F. candida*, an eyeless species can be seen in Figure 2C, which illustrates the external features of the body of *F. candida*. The lack of an external eye facet is readily apparent. We found no external eye structures on all ten *F. candida* specimens examined, confirming the “eyeless” status of this species.

### Ultraviolet (UV) light vs. white light

**Figure 3.**
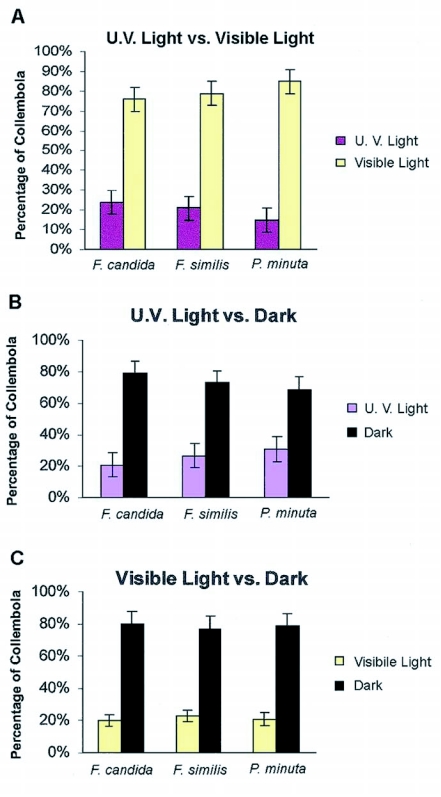
Graph (A) represents the average percentage of each species that preferred either white or (UV) light. Graph (B) illustrates the average percentage of each species that preferred (UV) light or dark and Graph (C) demonstrates the percentage of each species that preferred white light or dark. The number tested was (n = 28–74) in three different trials [p < 0.05].

Three different sets of ultraviolet (UV) light versus white light trials were performed for each species: *F. candida* (n = 34–57 per trial), *F. similis* (n = 24–61 per trial), and *P. minuta* (n = 26–71 per trial). Collembola were placed in the middle of the Petri dish at the beginning of each trial, and, at the end of each 30 min trial it was observed that a significant proportion were on the white light side of the choice-testing chamber. The results shown in Figure 3A confirm this observation: approximately 76% of the *F. candida*, [Heterogeneity *χ*^2^ = 0.45; p>0.05-all populations behaved similarly; pooled *χ*^2^ = 42.62; p < 0.001], 79% of the *F. similis*, [Heterogeneity *χ*^2^ = 1.70: p>0.05-all populations behaved similarly; pooled *χ*^2^ = 41.8; p<0.001] and 85% of *P. minuta*, [Heterogeneity *χ*^2^ = 2.40; p>0.05-all populations behaved similarly; pooled *χ*^2^ = 69.18; p<0.001] and were found in the white light side of the choice-testing chamber, thus avoiding the UV light.

### Ultraviolet (UV) light versus dark

*F. candida* (n = 33–73 per trial), *F. similis* (n= 44–59 per trial) and *P. minuta* (n = 43–66 per trial) were assayed for a choice between UV light and darkness in three different sets of trials. At the end of the 30 min trials, it was observed that they preferred the dark side of the choice-testing chamber. Figure 3B shows that for all three sets of trials a significant percentage preferred the dark side of the test chamber: 79%, of *F. candida* preferred the dark [Heterogeneity *χ*^2^ = 0.29; p>0.05-all populations behaved similarly; pooled *χ*^2^ = 71.14; p<0.001]; 73% of *F. similis* preferred the dark [Heterogeneity *χ*^2^ = 4.54; p>0.05-all populations behaved similarly; pooled *χ*^2^ = 67.12; p<0.001]; 69% of *P. minuta* preferred the dark [Heterogeneity *χ*^2^ = 1.50; p>0.05-all 3 populations behaved similarly; pooled *χ*^2^ = 89.62; p<0.001]. There were no significant differences in the behavior of Collembola among the different trials for each species but there was a significant percentage of all species that moved to the dark and away from UV light.

### White light versus dark

Three different sets of trials were performed with three different populations of *F. candida* (n = 33–63 per trial), *F. similis* (n = 28–47 per trial) and *P. minuta* (n = 41–58 per trial) to determine a preference between white light and darkness. At the end of 30 min trials, it was apparent that they preferred the dark side of the dish. Figure 3C illustrates that for all three sets of trials, a significant percentage preferred the dark: 80% of *F. candida* moved to the dark, [Heterogeneity χ^2^ = 0.24; p>0.05-all populations behaved similarly; pooled *χ*^2^ = 89.04; p<0.001]; 77% of *F. similis* moved to the dark, [Heterogeneity *χ*^2^ = 1.81; p>0.05-all populations behaved similarly; pooled *χ*^2^ = 39.77; p<0.001]; 79% of *P. minuta* moved to the dark, [Heterogeneity *χ*^2^ = 0.48; p>0.05-all populations behaved similarly; pooled *χ*^2^ = 55.44; p<0.001]. The results showed no significant differences in behavior of Collembola among group trials, yet a significant percentage moved to the dark and away from white light.

### Temperature choice - Test results

**Figure 4.**
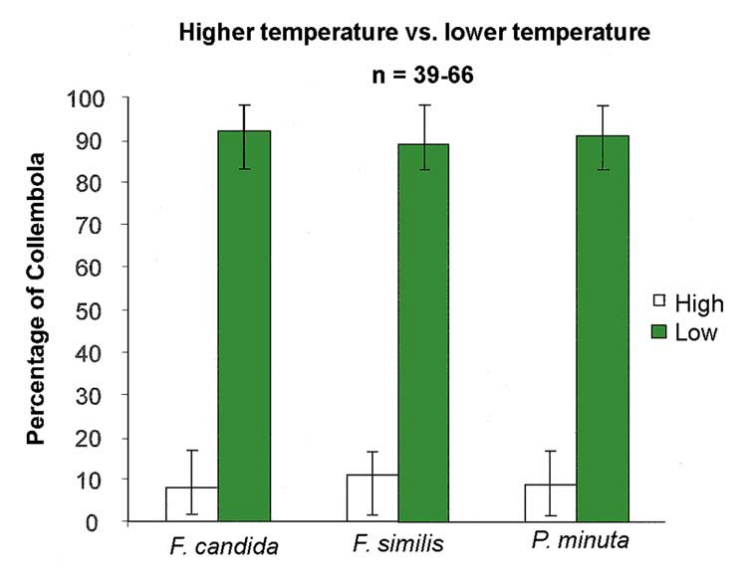
A histogram demonstrating the average percentage of each species of Collembola that preferred higher or lower temperatures. The average higher temperature was 30°C and the average lower temperature was 22°C. The number tested was (n = 39–66) in ten different trials [p < 0.05].

As a temperature difference was detected between each side of the choice-testing dish during the UV and white light choice-tests the three species were tested to determine they would make a choice between different temperatures. Figure 4 illustrates the mean percentage of collembolan species that preferred cooler or warmer temperatures of the choice-testing chamber. It can be seen in Figure 4 that a statistically greater percentage of Collembola [92% for *F. candida*, χ^2^ = 174.61: p< 0.001); 89% for *F. similis*, (*χ* ^2^ = 155.2: p< 0.001); and 91% for *P. minuta*, (*χ* ^2^ = 144.26: p<0.001)] chose the cooler temperatures over the warmer temperature. Thus, a temperature difference of approximately 8° C was sufficient to cause a significant alteration in preferential behavior of all species tested.

### Cool light versus cool dark choice - Test results

**Figure 5.**
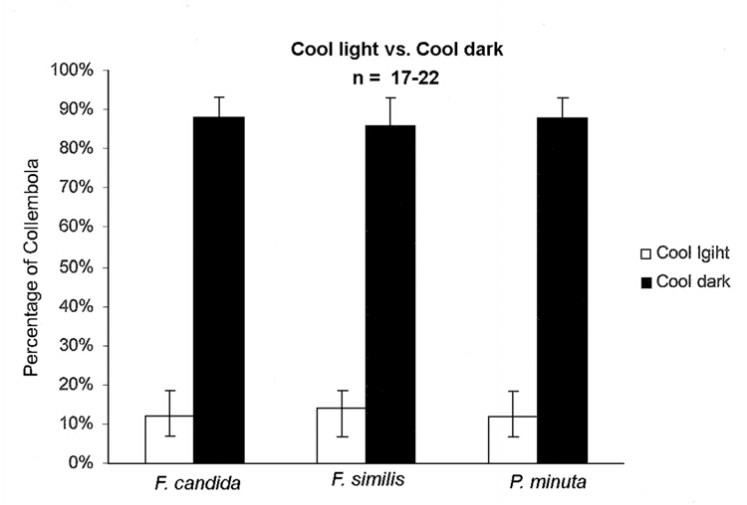
The average percentage of Collembola from each species that either chose cool light or cool dark. The number tested was (n = 17–22) in three different trials [p < 0.05].

A question arose as to whether the Collembola were merely moving away from white light because the light source used in the choice-tests produced heat (see previous results from UV light versus white light). To test the hypothesis that they will avoid white light regardless of temperature, the variable of temperature differences was eliminated. Collembola were tested for a choice between light and darkness, both with a mean temperature of 22 ± 0.5° C. Three different populations of each species were choice-tested in three separate trials. The results shown in Figure 5 illustrate that 85% of *F. candida* (n = 20–27 per trial) moved to the dark side, [Heterogeneity *χ* ^2^ = 0.16: p>0.05-all populations behaved similarly, pooled *χ* ^2^ = 40.13; p<0.001]; 84% of *F. similis* (n = 19–22 per trial) moved to the dark side, [Heterogeneity *χ* ^2^ = 0.08; p>0.05-all populations behaved similarly, pooled *χ* ^2^ = 31–741 p<0.001] and 82% of *P. minuta* (n = 17–22 per trial) moved to the dark side, [Heterogeneity *χ* ^2^ = 0.06; p>0.05-all populations behaved similarly, pooled *χ* ^2^ = 31.88; p<0.001]. The results demonstrated that a significant proportion from each species and in each trial moved to the dark side of the choice-testing chamber when given light as an alternative choice and both sides had the same temperature.

## Discussion

There have been few morphological and functional studies of collembolan eye physiology. While a previous phototactic study by Salmon and Ponge (1998) demonstrated that Collembola could distinguish among white light, blue light and dark, it was not clear whether they were able to detect UV light. Since, many other insects have been tested in response to UV light and there is suggested homology between the insect eye and collembolan eye, we hypothesized there might be a homology in function.

The original hypothesis of this study was that those species with eyes, *F. similis* (1 pair of eyes) and *P. minuta*, (8 pair of eyes), would detect UV light and respond to it given alternative choices of either white light or darkness. We further hypothesized that the eyeless species, *F. candida*, would not be able to make a distinct choice among UV, white light or dark.

Our assay tested the UV-detection capabilities of three species of Collembola by exposing them to different light conditions and observing their phototactic responses. The results demonstrated that Collembola chose white light when given ultraviolet light as an alternative choice, and chose darkness when given ultraviolet or white light as choices. The results also indicate that when exposed to UV light they preferred to avoid it and will move toward less optimal warm temperatures to avoid this visual condition.

These behavioral results confirm that Collembola, like many insects, have the capability to detect UV light. Since most insects have multiple forms of rhodopsin (Briscoe and Chittka 2001), we assume that Collembola must have homologous structures and signal transduction mechanisms to detect a similar range of light (Salcedo et al. 2003). There are approximately four types of rhodopsin that have evolved in insects: UV, blue, blue-green and long-wave length (Briscoe 2000). One would expect that collembolan photoreceptors would contain at least two forms of rhodopsin, UV and one, or more, rhodopsins to detect white light, which has a wide range of wavelengths. Judging by the results of Salmon and Ponge (1998), it is possible that the blue form of rhodopsin would play a strategic role in Collembola vision. It would be interesting to examine the molecule structure of these rhodopsin proteins to study the relationships between hexapod and crustacean clades.

The hypothesis that Collembola choose cool dark places was supported from our results. This is not a surprising since Collembola are diurnal soil dwelling arthropods, usually found in the soil, caves, lava tubes, leaf litter and under rocks (Chapman 1998). It is reasonable to hypothesize that such organisms would utilize some phototactic mechanism to detect UV and white light for orientation. It is also likely that they use light (UV or white) and dark as part of a circadian clock to help determine when to feed, reproduce or migrate.

Another possible explanation for the observed behavior is that Collembola use aggregation pheromones. While some species of Collembola have been reported to produce these odors (Verhoef et al. 1977, Christiansen et al. 1992, Manica et al 2001), we believe that our results were achieved irrespective of any pheromone release. First, other researchers (Heupel 2002, Dromph 2003) who studied avoidance behavior in Collembola showed that aggregation pheromones did not play a major role in their studies. Second, prior to the study, we ran a preliminary behavior assay testing each species in a white light chamber (no choice). Again, three replicates were done on each species. In every assay, the Collembola were equally distributed around the edge of the Petri dish chamber with no aggregates observed, suggesting that in white light they do not aggregate. Finally, between trials of each population group, the chamber bottoms were rotated 180° to avoid possible artifacts not connected to light differences. If the population discharged an aggregation pheromone after the first 30-minute trial, and then was tested again five minutes later, the collembolans would have theoretically aggregated in the pheromone side of the chamber which would now be in the lighted region. Thus, non-statistical significance would have been observed after testing for heterogeneity, which did not occur.

To examine the context of our behavioral assay, morphological studies of the eye structures were also performed. The aim of this part of the study was to correlate the external structure of the collembolan eyes with phototactic function by use of SEM analysis. The SEM analysis of the eyes of *P. minuta* suggests that the location of the collembolan eye fields is similar in location to the eye fields of insects such as ants, cockroaches, bees or *Drosophila melanogaster* (Gupta 1979; Chapman 1998). The external eye structures of the two species with eyes are similar and corroborate previous findings of Paulus (1970b, 1972b), Schaller (1970) and Barra (1971). Our SEM analysis demonstrates a possible pattern of facet reduction that can be elucidated by comparing between *P. minuta*, a species with eight pairs of eyes, and *F. similis*, with one pair of eyes. It can be perceived that the last eyes to become absent are located just behind the postantennal organ. Scanning electron microscopy of *F. candida* demonstrated that externally the eyes of these Collembola had no facets.

From the results of the behavioral assays and SEM analyses, it is obvious that the Collembola tested are making phototactic choices away from UV and white light and towards no light. Then, why do *F. candida*, an eyeless species, make the same phototactic choices as the other species when they have no obvious external eye structures? Based upon the SEM analysis, which shows the lack of facets, the “eyeless” *F. candida* may have internal photoreceptors or other photosensitive structures that elicit information regarding the visual spectrum. The underlying suggestion is that the eyeless species, *F. candida*, is not truly “eyeless” but rather are facetless, but yet are capable of discriminating among various light choices. In other “eyeless” studies performed with *Vanessa antiopa* (Lepidoptera), *Eristalis* (Diptera), *Dineutes* (Coleoptera), and *Cryptotympana* (Hymenoptera), when the eyes were blacked out on one side the insects would orient to light on the side where they still had sight. In the individuals that had their eyes totally black out they hardly moved in any direction or they did not move, (Chapman 1998). Apparently, they needed both the stimulus of light and functional photoreceptors for movement and orientation.

The phototactic results from our study supports the possibility of extra-ocular photoreceptor capability in *F. candida*. It is likely that *F. candida* lacks only external structures such as a lens and yet maintains some sort of internal photoreceptor cell or organelles that would allow it to detect both UV and white light. This “lensless” condition may have evolved in an organism that may require phototactic capabilities and structures, for orientation and directional cues. Since the faceted lens is generally used to focus light or images to the internal photoreceptor cells, organisms requiring light perception, but not resolution, may reduce lens number as it may well be beneficial to retain internal structures and not expend energy in the development and maintenance of external lenses.

In a study by Helfrich-Förster et al. (2001) it was shown that *Drosophila melanogaster* mutants that lack external compound eyes are still able to entrain to a circadian clock. In this study, putative extra-retinal eye structures known as Hofbauer-Buchner eyelets lie underneath the retina, and it is suggested this structure may play a role in the light input pathway in *Drosophila*. Paulus (1972c) described a vision organ found in the collembolan species *Tomocerus* that appeared to have migrated deep below the cuticle and lie near the esophagus. These structures appeared to be homologous to the crustacean frontal organ (Paulus, 1972c). There may be similar pathways or structures in the eyeless collembolan species, *F. candida*.

A putative, non-visual photoreceptor has been described in the carabid beetle, *Pachymorpha sexguttata* (Fleissner et al. 1993). This non-visual receptor is located within the optic lobe of the beetle. The optic lobe photoreceptor appears to play a role in the circadian circuitry of the beetle. *F. candida* may also have dermal phototactic capability. It has been shown that *Tenebrio* and *Papilio* larvae, which have ocular occlusions, can detect light and orient themselves accordingly due to epidermal cells that are apparently sensitive to light, (Chapman 1998). Here, the movements of pigment cells in the epidermal layers just beneath the cuticle have been observed, lending further support to the hypothesis that these insects can detect white light by other means besides the eye. In a recent study by Jordana et al. (2000), a new type of photoreceptor lacking external features has been described in the collembolan species, *Vesicephalus europaeus*. While this species has eight lenses per side, it also has an unusual, translucent interocular vesicle containing subcutaneous rhabdomes. In the case of *V. europaeus* and quite possibly *F. candida*, this type of internal photoreceptor structure may allow these Collembola to detect and distinguish UV and white light.

Complete histological studies of the eye region of these particular collembolan species are needed to investigate the possibility of internal photoreceptor structures located on the head of Collembola, or sensory ganglia that may be connected to the epidermal cells. In addition, it could be an interesting study to expose species from the collembolan Family Onichuriudae that are all eyeless, to the same behavioral assays described, to see if they make similar choices as the eyeless *F. candida*.
